# Dynamic Spectrum Sharing Based on Deep Reinforcement Learning in Mobile Communication Systems

**DOI:** 10.3390/s23052622

**Published:** 2023-02-27

**Authors:** Sizhuang Liu, Changyong Pan, Chao Zhang, Fang Yang, Jian Song

**Affiliations:** 1 Department of Electronic Engineering, Beijing National Research Center for Information Science and Technology (BNRist), Tsinghua University, Beijing 100084, China; 2 Peng Cheng Laboratory, Shenzhen 518055, China; 3 Shenzhen International Graduate School, Tsinghua University, Shenzhen 518055, China

**Keywords:** resource allocation, machine learning, dynamic spectrum access, deep reinforcement learning, deep Q-network

## Abstract

The rapid development of mobile communication services in recent years has resulted in a scarcity of spectrum resources. This paper addresses the problem of multi-dimensional resource allocation in cognitive radio systems. Deep reinforcement learning (DRL) combines deep learning and reinforcement learning to enable agents to solve complex problems. In this study, we propose a training approach based on DRL to design a strategy for secondary users in the communication system to share the spectrum and control their transmission power. The neural networks are constructed using the Deep Q-Network and Deep Recurrent Q-Network structures. The results of the conducted simulation experiments demonstrate that the proposed method can effectively improve the user’s reward and reduce collisions. In terms of reward, the proposed method outperforms opportunistic multichannel ALOHA by about 10% and about 30% for the single SU scenario and the multi-SU scenario, respectively. Furthermore, we explore the complexity of the algorithm and the influence of parameters in the DRL algorithm on the training.

## 1. Introduction

The rapid growth of the data traffic business has been facilitated by the advancement of mobile communication technologies. However, the limited availability of licensed spectrum with low utilization struggles to support such a vast scale of services [1]. As a result, the licensed spectrum used for data traffic is becoming overloaded. To address this issue, researchers have suggested utilizing unlicensed spectrum as a solution to alleviate the strain on licensed spectrum and improve network capacity. The upcoming generation of mobile communication systems will necessitate lower latencies, higher transmission rates, and more efficient utilization of the spectrum [2]. Therefore, an alternative way of allocating spectrum is needed to improve efficiency.

Since entering the 21st century, artificial intelligence technology has developed rapidly. Deep reinforcement learning (DRL) technology, as a branch of it, has also attracted the attention of researchers in various fields. Deep learning relies on artificial neural networks to create a complex connection between input and output data. The combination of deep learning’s feature extraction and perception abilities with the decision-making capabilities of reinforcement learning (RL) is achieved through the use of DRL, allowing agents to handle complex decision-making problems. In the training of RL, one commonly used value-based algorithm is Q-learning, but it may not be suitable for solving large-scale problems due to its inefficiency. The primary issues with Q-learning are that it requires a vast amount of storage capacity to keep track of the Q-values for each possible state-action pair, which renders it unfeasible for large-scale problems. Additionally, as the number of states in the problem increases, the algorithm’s efficiency deteriorates because of the infrequency of visiting certain states [3]. The DeepMind team introduced the Deep Q-Network (DQN) algorithm, which combines Q-learning with deep learning and achieved superior performance in the Atari game in 2013 [4].

The purpose of this study is to address the issue of spectrum sharing and power control in cognitive radio systems using DRL. The proposed algorithm uses DQN-based training with multiple agents for spectrum access strategy, modeling the allocation of multiple resources in communication systems as reinforcement learning, and designing a reward function for users. The training also incorporates freezing target networks and experience replay, and the complexity of the algorithm is analyzed. The simulation results demonstrate that secondary users (SU) are able to effectively learn how to access the spectrum through training with the proposed algorithm in both single SU and multiple SUs scenarios. The proposed method improves both the average reward and collision rate and is shown to be more effective than the opportunistic multichannel ALOHA (OMC-ALOHA) method and greedy method with prior information. In addition, experiments exploring the influence of parameters in the reward function and active rate of users are conducted. The contributions of this article are summarized as follows:

(1) The resource allocation problem in the CR system is modeled as a reinforcement learning task. SUs are represented as agents in this model and their chosen channels and transmission power are defined as actions. The reward is based on the quality of communication and potential collisions.
(2) we present a DRL-based algorithm for SUs to access the spectrum and control their transmission power in the CR system. The algorithm is based on the DQN algorithm and includes features, such as experience replay and freezing target networks. The artificial neural network structures used in the algorithm are DQN and DRQN. Through the use of a well-designed algorithm and network structure, users can learn and optimize their access strategies through training.
(3) Simulation experiments are implemented to compare the proposed algorithm with other policies and investigate the impact of various parameters such as the coefficients in the reward function and active rate. The results show that the proposed algorithm can effectively enhance system performance and reduce interference to the PUs.

We note that a shorter conference version of this paper [5] was presented in IEEE International Symposium on Broadband Multimedia Systems and Broadcasting 2021. Our initial conference paper did not show the detailed algorithms and analyze the complexity and did not finish the complete experiment of the parameters. Compared to the conference version, we have extended the simulation result section to compare the performance of the proposed algorithm with other existing methods and analyze the influence of the parameters in the algorithm on the result of the simulation.

The structure of the paper is as follows. The related work is introduced in Section 2, the system model is discussed in Section 3, the proposed DRL-based spectrum sharing method and training algorithm are outlined in Section 4, the simulation outcomes are shown in Section 5, and our conclusions are given in Section 6.

## 2. Related Work

There has been a significant amount of research on dynamic spectrum access technology in cognitive radio (CR) systems, with some studies using game theory to analyze spectrum sharing among users in the communication system [6]. Users in the communication system are modeled as players and their access strategies are analyzed. For example, in [7], a game-theoretic algorithm and utility function were presented for spectrum sharing in CR systems, while reference [8] proposed a method using game theory and the decision tree. A pricing strategy based on the Stackelberg game was suggested in another study [9] to improve spectrum sharing, and researchers developed an algorithm to minimize the cost of bandwidth allocation for primary users (PU) and SU [10]. Additionally, the Cournot model was applied to analyze PU spectrum allocation in CR systems [11].

Previous research has focused on the issue of spectrum handoff in CR networks, specifically addressing how SUs can access the spectrum without disrupting PUs. One solution that has been proposed is using a negotiation method based on fuzzy logic, as outlined in reference [12]. Another approach is utilizing particle swarm optimization (PSO) to minimize the time spent on spectrum handoff, as discussed in [13]. Additionally, a system using fuzzy logic controllers (FLCs) has been proposed to prevent unnecessary hand-offs in an LTE-Advanced system, as described in [14].

Applying DRL technology to manage resources in communication systems has proven to be a practical approach, with researchers using DRL in modern communication networks to improve the speed and performance of the algorithm [15]. Several studies have investigated models based on DQN for dynamic spectrum access in distributed communication systems [16,17,18], and a DQN-based power control method for cognitive radios to optimize communication quality through power adjustments was presented in [19]. The quality of communication was considered and a power control method was developed for a simple model with two users. In [20], the performance of DRL was tested in an interweave cognitive radio system, and literature [21] introduced a power control strategy based on DRL to enable SUs to adjust to the appropriate state in a limited time while avoiding interference with PUs. Another approach [22] used the asynchronous advantage actor-critic (A3C) and distributed proximal policy optimization (DPPO) for power adjustment to meet the quality of service requirements of each user. The spectrum was dynamically managed by a combination of DQN and evolutionary game theory in [23], and the double deep Q-network (DDQN) has also been applied to spectrum sharing [24,25]. In [26], a research project examined a spectrum management approach based on deep Q-learning, using the echo state network to implement Q-learning, enabling users to allocate the spectrum individually and intelligently. However, deep Q-networks are not suitable for problems with partial observation, so deep recurrent Q-networks (DRQN) have been implemented to handle the dynamic allocation of spectrum in discrete frequency channels [27,28,29]. Literature [30] considered the correlation between multiple channels. Besides distributive dynamic spectrum access (DSA), deep multi-user reinforcement learning is also applied to centralized DSA [31]. In addition, DRL is effective in DSA for vehicular ad hoc networks [32] and dynamic spectrum sensing and aggregation in wireless networks [33]. The channel access problem for cognitive radio-enabled unmanned aerial vehicles was studied with a DRL-based method [34]. Literature [35] noticed both the allocation of power and spectrum resources in the cognitive network, and reference [36] proposed a DSA based on traditional RL for the cognitive industrial Internet of Things (CIIoT). Despite their effectiveness, the majority of current solutions to the dynamic spectrum allocation problem have certain limitations. For instance, different from the actual system, users always need to transmit data in most studies [37].

## 3. System Model

We consider a cognitive radio system with *M* PUs and *N* SUs, each of which has a transmitter and receiver. PUs have high priority in the system and own the channels, while SUs can only access a channel for data transmission if it is not currently occupied by a PU. It is assumed that each PU in the system has its own channel, and can either be using a channel or not using one. The state of each PU is determined by the Markov process’s state transition matrix, which specifies the probabilities of changing from one state to another. Each SU has a specified likelihood of being active and needing to transmit, and when inactive, it takes no action. The SU’s power level is chosen from a pre-defined set of *L* options, and the locations of transmitters and receivers are distributed randomly within a certain area. The goal of the study is to allow SUs to use idle channels for communication when active, while minimizing power usage and meeting required transmission quality standards, without affecting the communication of PUs.

**Figure 1:**
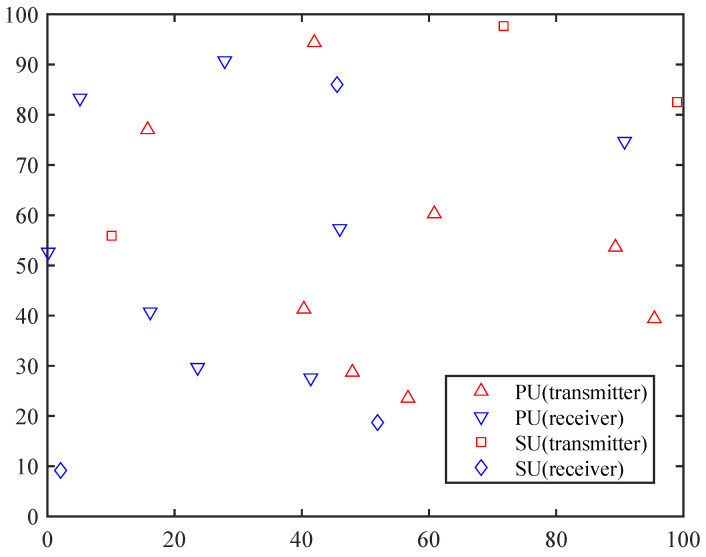
Positions of SUs and PUs.

SUs gather information about the channels by observing them in each time slot, including whether the current channels are occupied by PUs and the communication quality recorded by the receivers in that time slot. Based on the learning strategy, each SU makes a decision on whether to select a channel and transmission power or not select a channel at all. The state of the channels changes in the next time slot, and if the SU is active, the chosen channel and power level are used to transmit data. If the SU is inactive during the next time slot, it does not utilize any channel. The receivers of SUs record the signal-to-interference-plus-noise ratio (SINR), and the reward assigned to the SUs by the system is based on their SINR values and transmission powers. Figure 1 illustrates a sketch map showing the positions of transmitters and receivers with M=8 and N=3.

The channel capacity is the deterministic factor for the quality of communication in the system’s optimization objective. It represents the maximum rate of information that can be transmitted without error. The calculation of channel capacity can be done with SINR. In this paper, the SINR at the receivers of SUs is calculated using the WINNER II channel model [38], which is used to determine the channel gain from transmitters to receivers. WINNER II channel is a widely recognized standard in the area of wireless communication, and can be used to predict the performance of multi-channel wireless systems in different environments, considering various scenarios [39], and therefore is adopted as the channel model in the simulation. If the *i*-th SU selects a channel that is not occupied by the PU, the SINR value at the *i*-th secondary receiver is determined by [1,19]

(1) $${\mathrm{SINR}}_{i}=\frac{p_{i}\left|h_{ii}^{2}\right|}{\sum_{k\neq i}p_{k}\left|h_{ki}^{2}\right|+BN_{0}},i=1,2,\cdots ,N,$$

where the channel gain from the *i*-th transmitter to the *i*-th receiver and from the *k*-th transmitter to the *i*-th receiver are represented by $h_{ii}$ and $h_{ki}$, respectively. The transmission powers of the *i*-th and *k*-th SUs are denoted by $p_{i}$ and $p_{k}$, respectively. The bandwidth of the channel and noise spectral density are represented by *B* and $N_{0}$, respectively.

The training algorithm proposed in this paper is based on the DQN algorithm, which combines deep learning and Q learning. Q learning is a reinforcement learning algorithm, which is independent of the model. It judges the current optimal action by calculating the value function of each action to complete the decision. In the DQN algorithm, the Q table for querying value functions based on actions and states in Q learning is replaced by the artificial neural network, so as to alleviate the problem that the Q table is too complex when the number of states and behaviors is large in Q learning and improve the learning efficiency.

## 4. DRL-Based Spectrum Sharing Method

### 4.1. Reinforcement Learning Model

**Figure 2:**
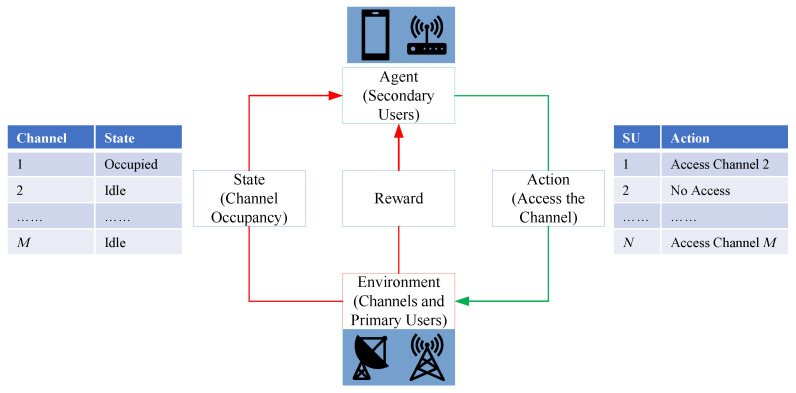
Principle of the reinforcement learning techniques.

In the process of reinforcement learning, the agents learn how to map the current environment information to their behavior in the training process. Through repeated attempts, the agents can independently find the most valuable action with the highest reward. These actions will determine the reward of the agent, affect the environment at the next moment, and indirectly affect the subsequent reward [40]. Figure 2 shows the basic principle of reinforcement learning. In order to transform the problem of resource allocation in the unlicensed spectrum into the problem of reinforcement learning, it is necessary to clarify the specific meanings of agent, environment, state, action, and reward in this problem.

In this study, SUs are referred to as agents, and their actions represent the channel and power usage choices they make. The reward function is on the basis of the quality of communication, taking into account whether collisions occur and the SU’s power usage. If a SU accesses a channel that is currently occupied by a PU, a collision occurs, resulting in a negative reward for the SU. If a SU does not utilize any channel, the reward assigned to it is 0. The reward in other situations is largely determined by the capacity of the channel, which is related to the SINR. When there is only one available power level (L=1), the reward is determined according to [1]

(2) $$r_{s}=\left\{\begin{array}{cc} 0 & ,\mathrm{no}\;\mathrm{channel}\;\mathrm{access},\\ -C & ,\mathrm{collision}\;\mathrm{with}\;\mathrm{PU},\\ \log_{2}\left(1+\mathrm{SINR}\right) & ,\mathrm{other}\;\mathrm{cases}\;, \end{array}\right.$$

where *C* is a positive constant. When multiple available transmit powers are considered, the reward function is designed as

(3) $$r_{m}=\left\{\begin{array}{cc} 0 & ,\mathrm{no}\;\mathrm{channel}\;\mathrm{access}\;\mathrm{or}\\ \; & \;\;\mathrm{SINR}\;\mathrm{is}\;\mathrm{not}\;\mathrm{up}\;\mathrm{to}\;\mathrm{standard},\\ -C_{1} & ,\mathrm{collision}\;\mathrm{with}\;\mathrm{PU}\;,\\ C_{2}-C_{3}\log_{2}P & ,\mathrm{SINR}\;\mathrm{is}\;\mathrm{UP}\;\mathrm{to}\;\mathrm{standard}, \end{array}\right.$$

where positive coefficients $C_{1}$, $C_{2}$, $C_{3}$, and transmission power *P* determine the reward. The trend and relative value of the reward are more significant than its absolute value.

### 4.2. Deep Q-Network and Deep Recurrent Q-Network

**Figure 3:**
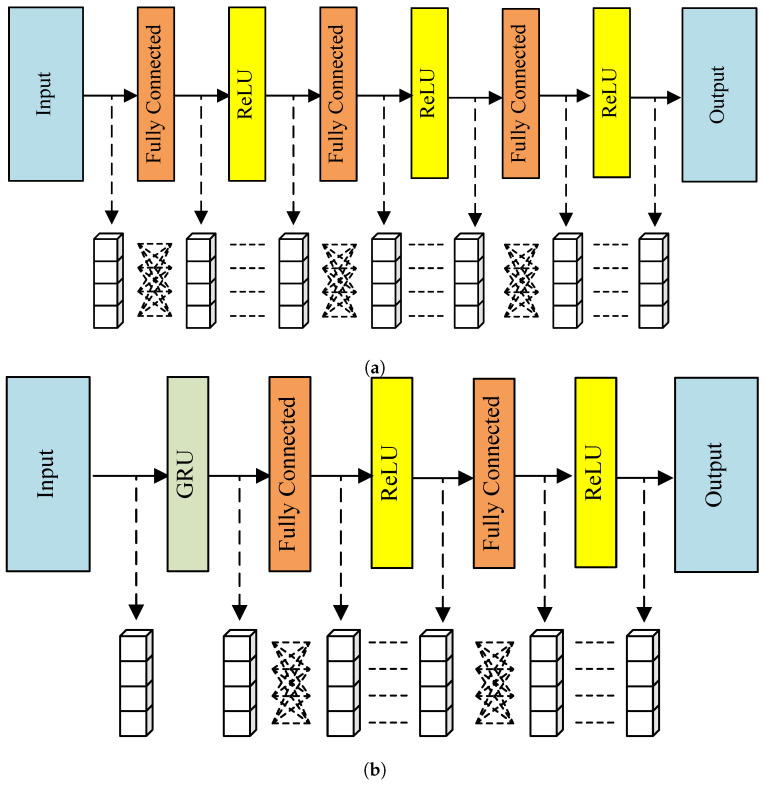
Illustration of the adopted network structures. (**a**) Illustration of the adopted DQN structure. (**b**) Illustration of the adopted DRQN structure.

The training algorithm in this study utilizes the state of channel usage as an input for the DQN, which outputs Q values for the actions and includes fully connected layers. There is a separate DQN for each SU in the system. The optimization method chosen is a gradient descent-based algorithm, such as stochastic gradient descent (SGD) or mini-batch gradient descent (MBGD). Once training is complete, the final decisions on which channel to access and which transmission power to use for SUs are determined by

(4) $$a_{n}=\underset{a}{\arg\max}Q\left(s,a\right),$$

where Q(s,a) is the output for a given state *s* and action *a*. The complete extraction and integration of information from input data can be achieved through the fully connected layer in the neural network. The ability to learn more intricate relationships between input and output data is facilitated by an increase in the number of layers. Nonetheless, a higher layer count demands a larger amount of training data and more calculations. Thus, the network architecture selected in the DQN method has three fully connected layers. In the simulation for the DRQN model, to provide the users with memory capabilities to process sequences with time information, the DQN is replaced with DRQN, which includes a gated recurrent unit (GRU) layer in addition to fully connected layers. The GRU layer allows the network to avoid the problem of long-term dependency [41] in traditional recurrent neural network (RNN) structures, and it is a more straightforward neural network architecture compared to long short-term memory (LSTM) [42]. Figure 3a,b show the complete network structures of the DQN and DRQN, respectively, and the effectiveness of both DQN and DRQN structures is assessed through the simulations.

During the training of DQN, the weights of the network are randomly initialized. Each SU observes and adopts the ϵ-greedy method [1] to select an action as

(5) $$a_{n}=\left\{\begin{array}{cc} \underset{a}{\arg\max}\;Q\left(s,a\right), & \mathrm{with}\;\mathrm{probability}\;-\epsilon ,\\ \mathrm{random}\;\mathrm{action,}\;, & \mathrm{with}\;\mathrm{probability}\;\epsilon , \end{array}\right.$$

where ϵ is a parameter that ranges from 0 to 1. The next state and the reward are calculated by the system and the results are stored. After sufficient training data are gathered, the weights of the neural networks are updated using the optimization method. The freezing target network technique [43] is used to enhance the training effect and minimize oscillation and the loss function is described by [4]

(6) $$L\left(\theta \right)=E[{\left(r+\gamma Q^{*}\left(s^{\prime },a^{\prime }\right)-Q\left(s,a\right)\right)}^{2}],$$

where θ are the weights of DQN, the discounted rate of the future reward is represented by γ. $Q^{*}$ and *Q* are the target network and the estimation network, respectively. The estimation network *Q* is updated in each iteration, but the target network $Q^{*}$ remains unchanged until it is occasionally updated with the value of *Q*. The order of the training data is shuffled and the experience replay technique is utilized to break the correlations between training data and improve the efficiency of training. The replay memory [3] is used to store the agents’ experiences for future training, improving the utilization of the training data.

During the training of DRQN, the sequential information is preserved when updating the weights of the neural network, rather than shuffling the order. The neural network processes continuous sequences of input for calculation. Due to the characteristics of RNN, it is impossible to perform the gradient descent on the data of one time slot alone when updating parameters. Instead, it is necessary to calculate the gradient of each weight of the network according to the backpropagation through time algorithm (BPTT) [44] with a sequence of a certain length as the sample, and update the parameters. In this process, the gradient of the GRU part needs to be calculated iteratively in a specific time sequence. Since the hidden state in the GRU network needs to be updated with every data input, DRQN calculation is also required even when the SU is inactive. The other training procedures of DRQN are similar to those for DQN.

### 4.3. DRL-Based Training Algorithm

The complete training algorithms of the DQN method and the DRQN method are shown in Algorithms 1 and 2, respectively. *S* is the current state of the system, $S_{n}$ is the next state of the system, *a* is the action selected by the SU at the next time slot, *r* is the reward received by the SU at the next time slot, and active represents whether the SUs are active. ${\mathrm{DQN}}_{e}$ is the estimation network, and ${\mathrm{DQN}}_{t}$ is the target network. *T* is the length of the extracted sequence in the DRQN method. *E*, $B_{1}$, and $B_{2}$ are constants that control the number of training steps.
**Algorithm 1 **Training Algorithm of the DQN MethodInitialize the state of the system and the weights of ${\mathrm{DQN}}_{e}$ of each SU. ${\mathrm{DQN}}_{t}$ = ${\mathrm{DQN}}_{e}$.**for**episode=1,E**do**   **for** $i=1,B_{1}$ **do**     **for** n=1,N **do**        **if** ${\mathrm{SU}}_{n}$ is active in the next time slot **then**          Input *S* into the ${\mathrm{DQN}}_{e}$ of ${\mathrm{SU}}_{n}$ and obtain the output.          ${\mathrm{SU}}_{n}$ obtains $a_{n}$ according to the ϵ-greedy method.        **end if**     **end for**     The state of the system changes, calculate *r* and store a set of *S*, $S_{n}$, *a*, *r*.   **end for**   Randomly extract $B_{2}$ groups of data from the experience memory.   **for** $i=1,B_{2}$ **do**     **for** n=1,N **do**        **if** ${\mathrm{SU}}_{n}$ is active in the next time slot **then**          Input *S* in the *i*-th group of data to ${\mathrm{DQN}}_{e}$ and ${\mathrm{DQN}}_{t}$, and obtain the outputs.          Calculate the gradient and update the weights of ${\mathrm{DQN}}_{e}$.        **end if**     **end for**     Every certain number of samples, let ${\mathrm{DQN}}_{t}$ = ${\mathrm{DQN}}_{e}$.   **end for****end for**

**Algorithm 2 **Training Algorithm of DRQN Method
Initialize the state of the system and weights of ${\mathrm{DRQN}}_{e}$ of each SU. ${\mathrm{DRQN}}_{t}$ = ${\mathrm{DRQN}}_{e}$.
**for** episode=1,E **do**
   **for** $i=1,B_{1}$ **do**     **for** n=1,N **do**        **if** ${\mathrm{SU}}_{n}$ is active in the next time slot **then**          Input *S* into the ${\mathrm{DRQN}}_{e}$ of ${\mathrm{SU}}_{n}$ and obtain the output.          ${\mathrm{SU}}_{n}$ obtains $a_{n}$ according to the ϵ-greedy method.        **end if**     **end for**     The state of the system changes, calculate *r*, and store a set of *S*, $S_{n}$, *a*, *r*.   **end for**   Randomly extract $B_{2}$ sequences. Each sequence is composed of consecutive *T* groups of data.   **for** $i=1,B_{2}$ **do**     **for** n=1,N **do**        **for** t=1,T **do**          Input the *S* in the *i*-th extracted sequence to ${\mathrm{DRQN}}_{e}$ and ${\mathrm{DRQN}}_{t}$, and obtain the outputs.          Record relevant information for subsequent calculation of gradient.        **end for**        Calculate the gradient based on recorded information and update the weights of ${\mathrm{DRQN}}_{e}$.     **end for**     Every certain number of samples, let ${\mathrm{DRQN}}_{t}$ = ${\mathrm{DRQN}}_{e}$.   **end for**
**end for**



The space complexity of the DQN and DRQN algorithm mainly comes from a large number of training data stored in the experience memory. Each group of data includes five items (*S*, $S_{n}$, *a*, *r*, and active). Among them, *S* and $S_{n}$ contain the occupancy information of *M* channels and, therefore, have a space complexity of O(M). *a*, *r*, and active are generated for each SU, so they have a space complexity of O(N). In summary, the total space complexity of Algorithms 1 and 2 is O(M+N).

In the process of generating data, the generation of each group of data requires three matrix multiplication operations and three ReLU operations for each SU. The time complexity of generating data for each SU is $O(M^{2}L^{2})$, where *L* is the number of selectable power levels. The time complexity of the gradient calculation process of Algorithm 1 when the weights of a SU are updated is $O(M^{3}L^{2})$. Algorithm 1 has a time complexity of $O(M^{3}NL^{2})$ while the total time complexity of Algorithm 2 is $O(M^{2}NL^{2})$.

## 5. Simulation Results

### 5.1. Performance under a Different Number of Users

**Figure 4:**
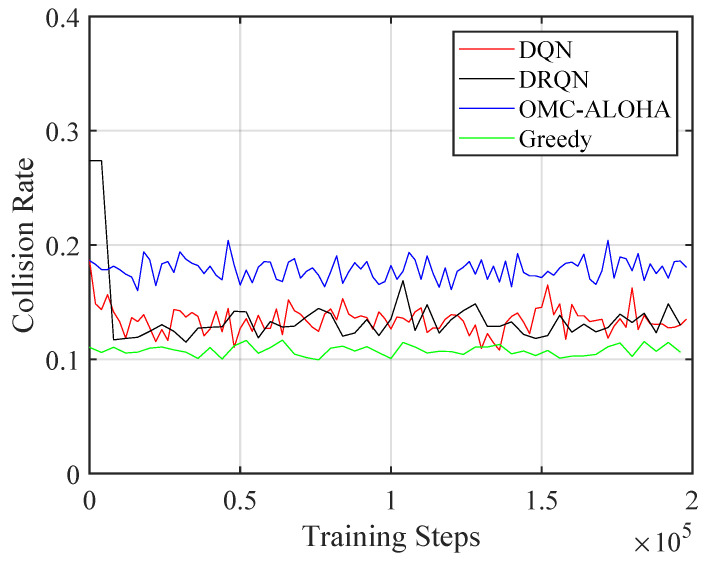
The collision rate of the proposed method with different training steps (single SU).

**Figure 5:**
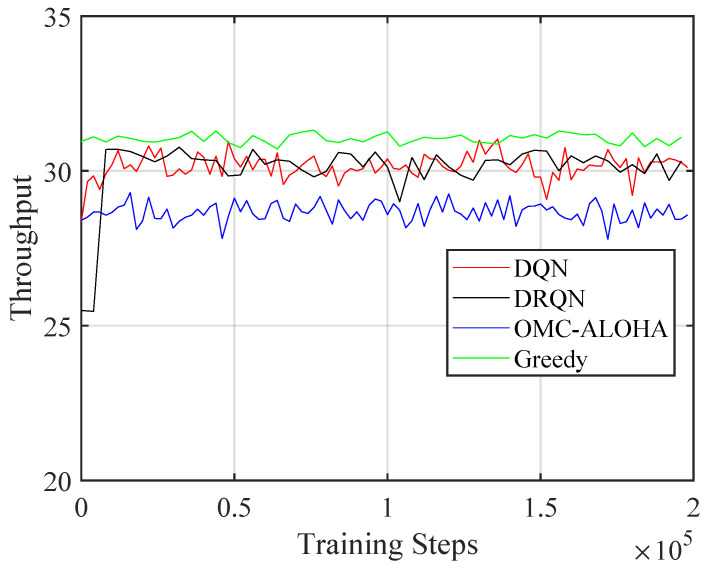
The throughput of the proposed method with different training steps (single SU).

**Figure 6:**
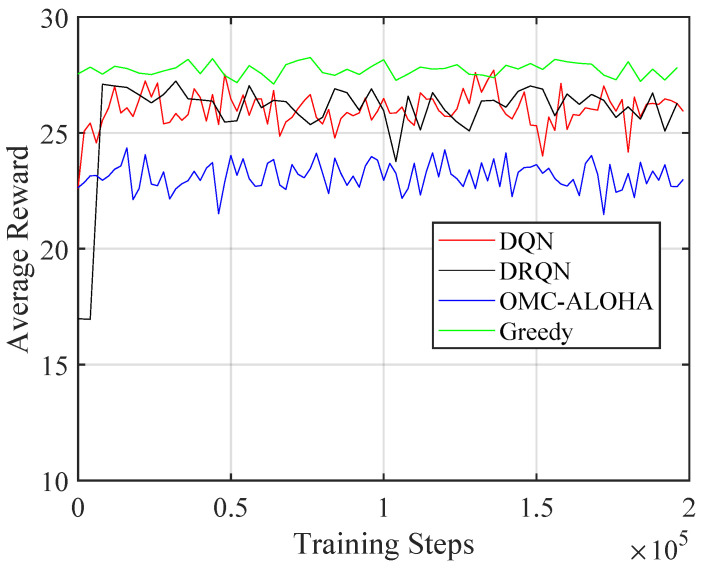
The average reward of the proposed method with different training steps (single SU).

**Figure 7:**
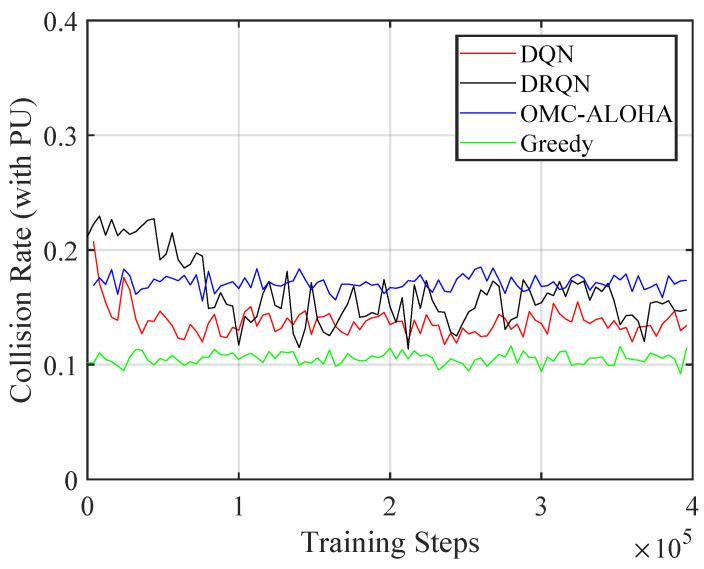
The collision rate of the proposed method with different training steps (multiple SUs).

**Figure 8:**
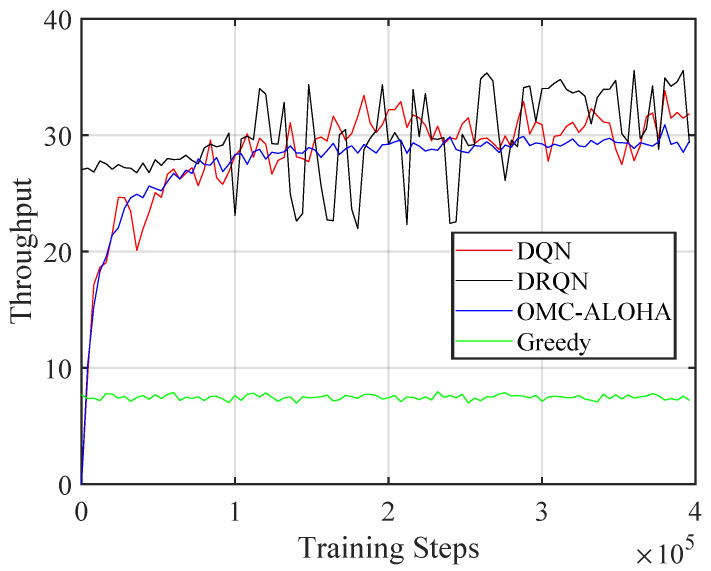
The throughput of the proposed method with different training steps (multiple SUs).

**Figure 9:**
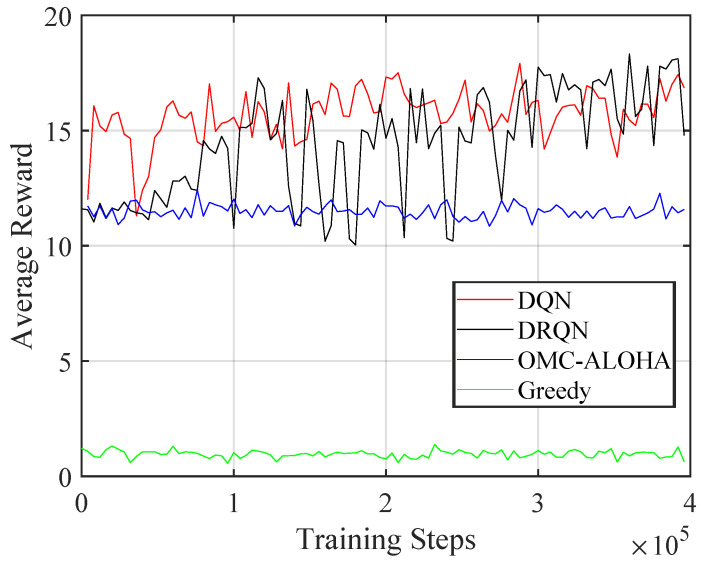
The average reward of the proposed method with different training steps (multiple SUs).

Figure 4, Figure 5, Figure 6, Figure 7, Figure 8 and Figure 9 display the results of simulations that demonstrate the changes in collision rate and average reward during the training of DQN and DRQN. The performance of the DRL-based methods is compared to the greedy method with prior information and OMC-ALOHA scheme in [45]. In each time slot, SUs access the channel that they expect will yield the highest reward, based on the prior information about PUs’ state transition matrix in the greedy method.

From Figure 4, Figure 5 and Figure 6, the number of PUs is set to 15 and there is a single SU. The active rate of the SUs is set to 1, and the SU uses a fixed transmission power. Simulation parameters are listed in Table 1. The reward value of SU is calculated using (2). New training data are created based on (5) and stored in the replay memory during each iteration. The weights of the network are updated using randomly selected sequences from the replay memory, which are also used to calculate the collision rate and the average reward.

Training resulted in a decrease in the collision rate and an increase in throughput and the average reward, demonstrating the effectiveness of the proposed method. The collision rate significantly decreased at the start of training and then gradually stabilized. The throughput and the reward increased during training and converged, in contrast to the collision rate, which decreased over time. There was no notable performance difference between the DQN and DRQN structures. DRQN reached its optimal performance at a slower pace compared to DQN. Both outperformed the OMC-ALOHA method in terms of collision, throughput, and reward, and were comparable to the greedy method. This is because the greedy method has access to prior information that the DRL method lacks, and is theoretically the optimal solution in the case of a single SU. As a result, the DRL method can be seen as delivering near-optimal results in situations where there is only one SU and no prior information regarding the PU is available. The greedy method—being the optimal solution for a single SU—makes the result of the simulation satisfactory. The SU is able to acquire the strategies that maximize the reward while minimizing collisions.

From Figure 7, Figure 8 and Figure 9, the number of PUs is set to 8 and there are 4 SUs, each with 3 available power levels. The active rate of the SUs is set to 0.3. Simulation parameters are listed in Table 2. The reward calculation for the case of multiple SUs is determined by (3). The results indicate that compared to the OMC-ALOHA and greedy methods, the proposed methods can achieve a higher reward in this scenario. When the greedy algorithm is used, all SUs prefer to choose the same channel that they believe is the best, leading to an increase in collisions between SUs and a decrease in average reward. The OMC-ALOHA method achieves a throughput close to the DQN and DRQN methods, but receives a lower reward due to a higher collision rate and a lower communication quality compliance rate. The DRQN method slightly surpasses the DQN method in terms of collision rate and throughput, resulting in roughly comparable overall performance. The DQN structure also converges faster than the DRQN structure in this scenario. The SUs can develop strategies that enable them to avoid collisions with PUs and other SUs while accessing channels with appropriate transmission power in both scenarios. The simulation findings indicate that in the system model established in the paper, both the DQN and DRQN algorithms produce comparable outcomes after convergence, however, the convergence pace of DRQN is slower compared to that of the DQN algorithm. This slower rate may be due to the intricate structure of the GRU layer in DRQN. Nonetheless, in a more complex system model, the DRQN algorithm may surpass the performance of a fully connected network by learning the sequences.

### 5.2. Parameters in the Reward Functions (2) and (3)

In reinforcement learning, the design of the reward function directly affects the learning objectives of agents and has an important impact on the results. Therefore, this section adjusts the coefficients in the reward function and observes the change. Table 3 shows the reward after 200,000 training steps with different coefficients *C* in (2) with M=10, N=1, and L=1. The order of magnitude of *C* should not differ too much from $\log_{2}\left(1+\mathrm{SINR}\right)$, so C is set between 3 and 300.

The reduction of *C* means that the loss caused by the risk of collision with PU is reduced. The system encourages users to access as much as possible. However, the result does not increase significantly when C=3. The possible reason is that the channel is idle for a system with only one SU. As a consequence, the SU always tends to access even if C=30. When the collision penalty is large enough, the reward is close to zero. The system is affected by the excessive value of *C*, and the high cost of collision risk causes the SU to tend not to access. In this case, the utilization rate of the spectrum is extremely low, which is a phenomenon that needs to be avoided. Therefore, it is necessary to set an appropriate *C* value.

For the case of multiple SU, we explore the impact of $C_{1}$ and $C_{2}$ in (3) on the simulation. As mentioned earlier, the relative value of the reward is more important than the absolute value. Therefore, we only need to focus on the ratio of $C_{1}$ to $C_{2}$. Fix the value of $C_{1}$ as 25, and only adjust the value of $C_{2}$. The reward and collision rates are represented in Table 4 and Table 5 when M=8, N=3, and L=3.

Increasing the value of $C_{2}$ is equivalent to reducing the penalty of collision with PU and encouraging SU access. Similar to the situation of C=3 in Table 3, the system resources are abundant, so a large $C_{2}$ has little influence on the collision rate. The rewards in all of the methods rise as $C_{2}$ rises. When the value of $C_{2}$ is low, the excessive penalty makes the DRL-based method unable to work properly and has no advantage over the random and greedy methods. The algorithm can achieve effect only when the proportion between parameters is appropriate.

### 5.3. Active Rate

Unlike most related works, it is assumed that SU does not always need to communicate in this paper, so it is important to explore the impact of the SU’s active rate on experimental results. When the number of PU is more than SU, the system is idle and the spectrum resource is abundant. The change in the active rate has little influence on the experimental results. Therefore, we consider that the system is in a state of resource shortage; that is, there is fewer PUs than SUs in the system (M=3, N=6, L=3). The reward after 200,000 steps under different active rates is shown in Table 6.

When the active rate of SUs is 1, the available channels are insufficient to meet users’ needs. After DRL-based training, some SUs will give up access and the others will continue to try to access, making the average reward slightly greater than 0. The random method and greedy method obtain negative rewards due to high collision. Under various activity rates, the DQN method and DRQN method have significantly better performance than the random method and greedy method. In the extreme case of spectrum shortage, the proposed method can still use communication resources for data transmission as much as possible.

## 6. Conclusions

In this paper, we deal with the resource allocation challenge in CR systems through DRL. In the system, SUs are modeled as agents, and their selection of channels and utilization of transmission power are deemed as their actions. The reward is designed based on the communication quality and whether collision exists. To enhance access to the spectrum and manage transmission power in CR systems, a DRL algorithm is put forward, which blends the DQN algorithm with techniques, such as freezing target networks and experience replay. The DQN and DRQN neural network structures are employed in the algorithm to improve access strategies. We conducted simulation experiments to verify the performance of the algorithm and examine the impact of factors, such as the coefficients of reward and active rate. The results show that the proposed algorithm can significantly enhance system performance while decreasing interference to the PUs. The proposed method surpasses OMC-ALOHA in the reward by approximately 10% and 30% in the single SU scenario and the scenario with multiple SUs, respectively. The algorithm complexity was also examined, and the influence of parameters on experimental results was investigated. The results indicate that the method is effective under diverse active rates of SUs when reward function coefficients are set properly.

## Figures and Tables

**Table 1:** Parameters of the first simulation (Single SU).

Parameter	Value
Number of PU	15
Number of SU	1
Active Rate	1
Selectable Transmission Power	20 mW
Learning Rate	$10^{-6}$
Discounted Rate (γ)	0.9

**Table 2:** Parameters of the second simulation (Multiple SUs).

Parameter	Value
Number of PU	8
Number of SU	4
Active Rate	0.3
Selectable Transmission Power	1 mW, 10 mW, 100 mW
Learning Rate	$10^{-6}$
Discounted Rate (γ)	0.9

**Table 3:** The reward after 200,000 steps under different *C* (Single SU).

Method	C=3	C=30	C=300
DQN	28.7938	23.8333	−0.1785
DRQN	28.4703	25.0642	0
Random	23.6995	20.0518	−28.3294
Greedy	29.1707	25.9507	0

**Table 4:** The reward after 200,000 steps under different $C_{2}$ (multiple SUs).

Method	$C_{2}=3$	$C_{2}=30$	$C_{2}=300$
DQN	−6.2809	15.2238	223.8512
DRQN	−3.7978	16.9011	246.8618
Random	−4.9356	4.9345	161.5352
Greedy	−3.9868	5.2668	135.2807

**Table 5:** The collision rate after 200,000 steps under different $C_{2}$ (multiple SUs).

Method	$C_{2}=3$	$C_{2}=30$	$C_{2}=300$
DQN	20.6760% (with PU)	18.1336% (with PU)	21.4618% (with PU)
	12.9720% (with SU)	2.9058% (with SU)	4.3354 (with SU)
DRQN	17.4286% (with PU)	16.8952% (with PU)	15.4653% (with PU)
	0 (with SU)	0 (with SU)	0 (with SU)
Random	17.8571% (with PU)	21.0887% (with PU)	18.2176 (with PU)
	3.9442% (with SU)	5.1209% (with SU)	2.0970% (with SU)
Greedy	10.5238% (with PU)	11.8952% (with PU)	14.6789% (with PU)
	63.7143% (with SU)	59.7984% (with SU)	46.2647% (with SU)

**Table 6:** The reward after 200,000 steps under different active rates (M=3, N=6, L=3).

Method	Active Rate = 1	Active Rate = 0.5	Active Rate = 0.2
DQN	4.0523	5.9819	9.6539
DRQN	2.0296	6.8767	9.5976
Random	−2.2040	3.7478	5.1396
Greedy	−2.9390	−2.5156	−2.5097

## Data Availability

Not applicable.
